# A Text-Book of Pharmacology and Therapeutics

**Published:** 1900-03

**Authors:** 


A Text-Book of Pharmacology and Therapeutics, or the Action of Drugs in Health and Disease.—By Arthur R. Cushney, M. A., M. D., Professor of Materia Medica and Therapeutics in the University of Michigan, etc. Illustrated with forty-seven engravings. 730 pages. Price, $3.50. Lea Brothers & Co., Philadelphia and New York. 1899.

It can be said of the above work that it is not a compilation, a "literary heredity," so to speak. The author has made every statement, after investigating the subject for himself, laboratory and clinical experimentation having been made the basis. He says in the preface: "My object has been to bridge over the hiatus which exists between the phenomena occurring in the normal organism and those which are elicited in the therapeutic use of drugs, to show how far the clinical effects of remedies may be explained by their action on the normal body, and how these may in turn be correlated with physiological phenomena."

The author has mentioned some drugs to condemn them. He might have enlarged his list, and still remained in good repute with most physicians. It is a fancy entertained by some chemical houses that new drugs and compounds are always the best, and it shows up-to-dateness to employ them. Cushney's book shows independent investigation, and is characterized by conservative yet fearless statements.

The work, however, is more especially devoted to pharmacology than to therapeutics. T. J. B.

